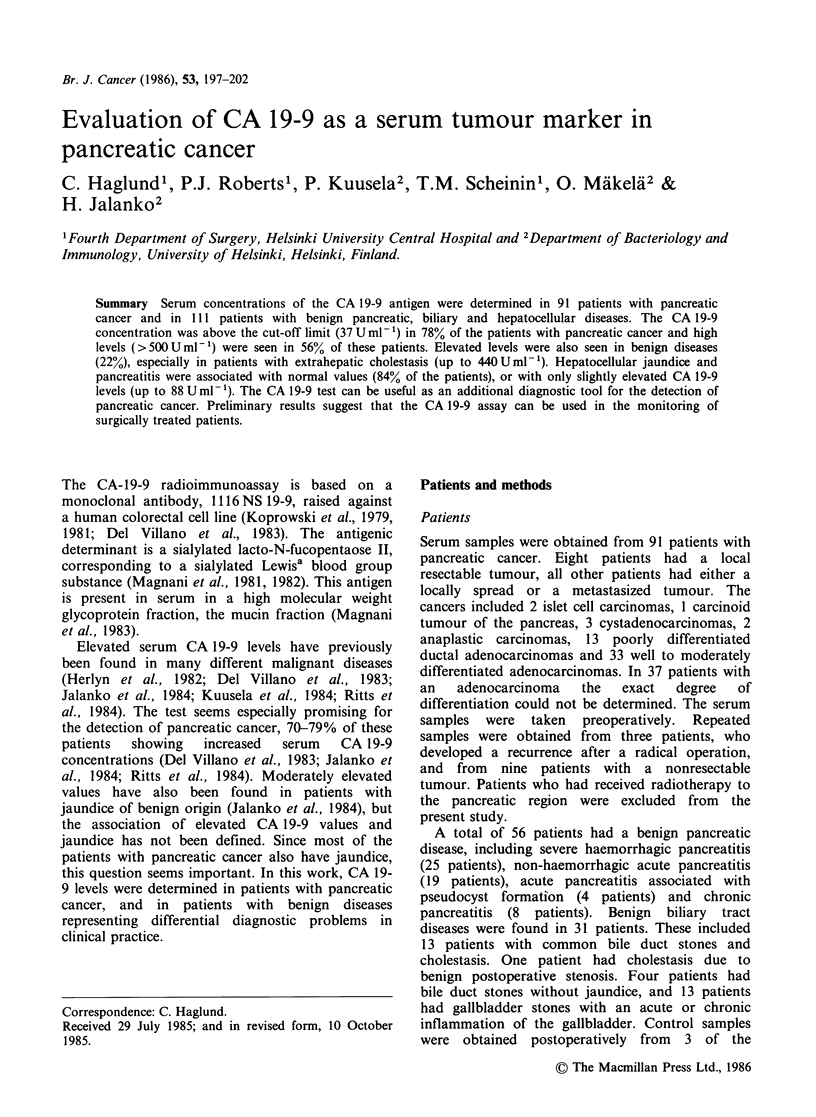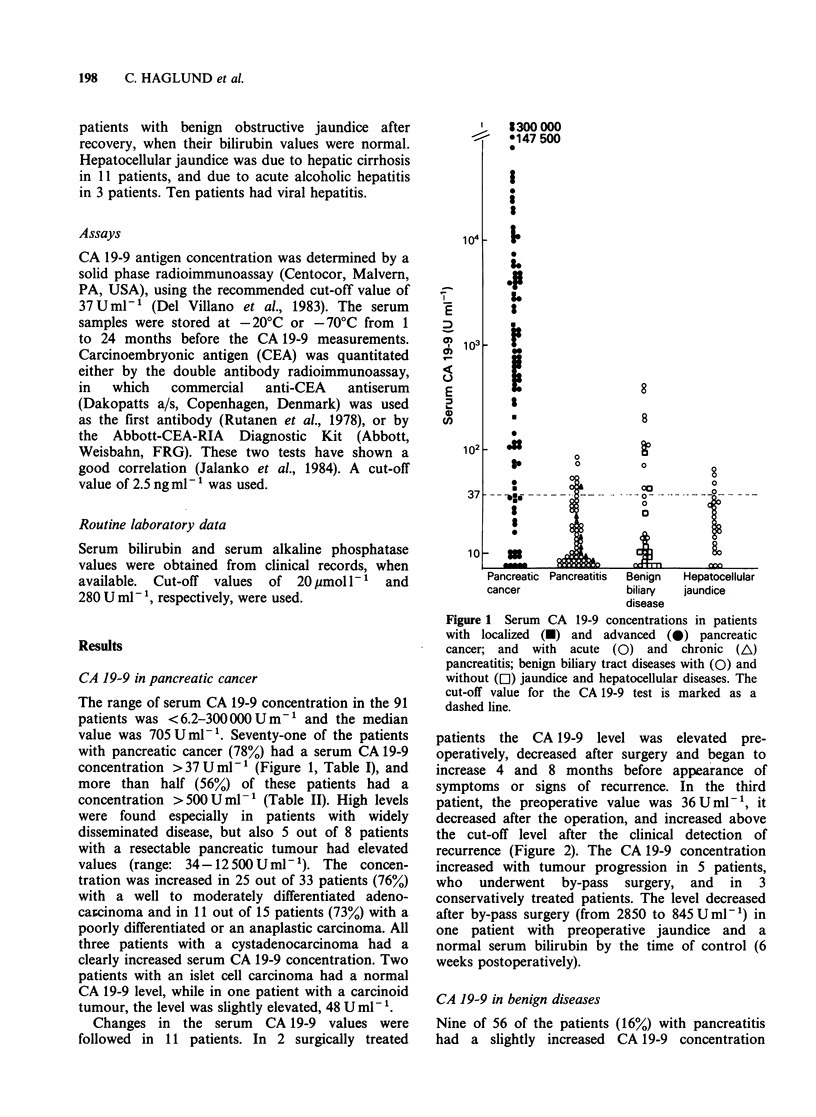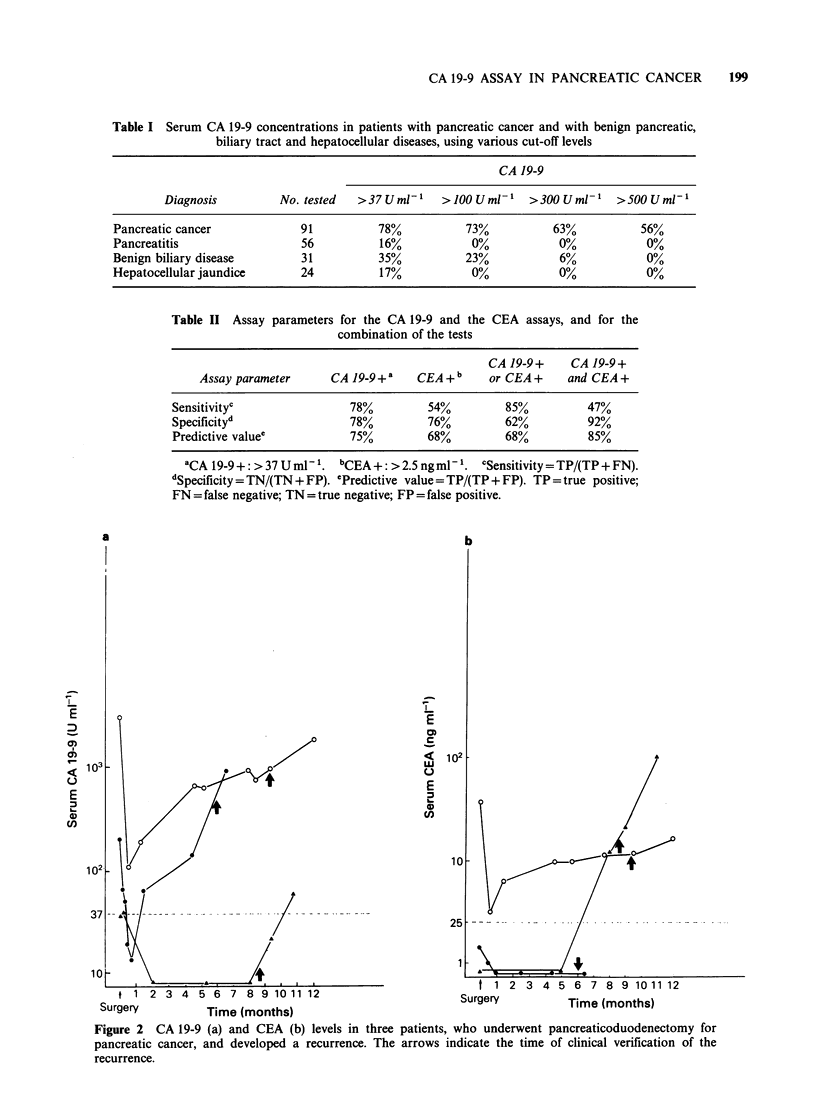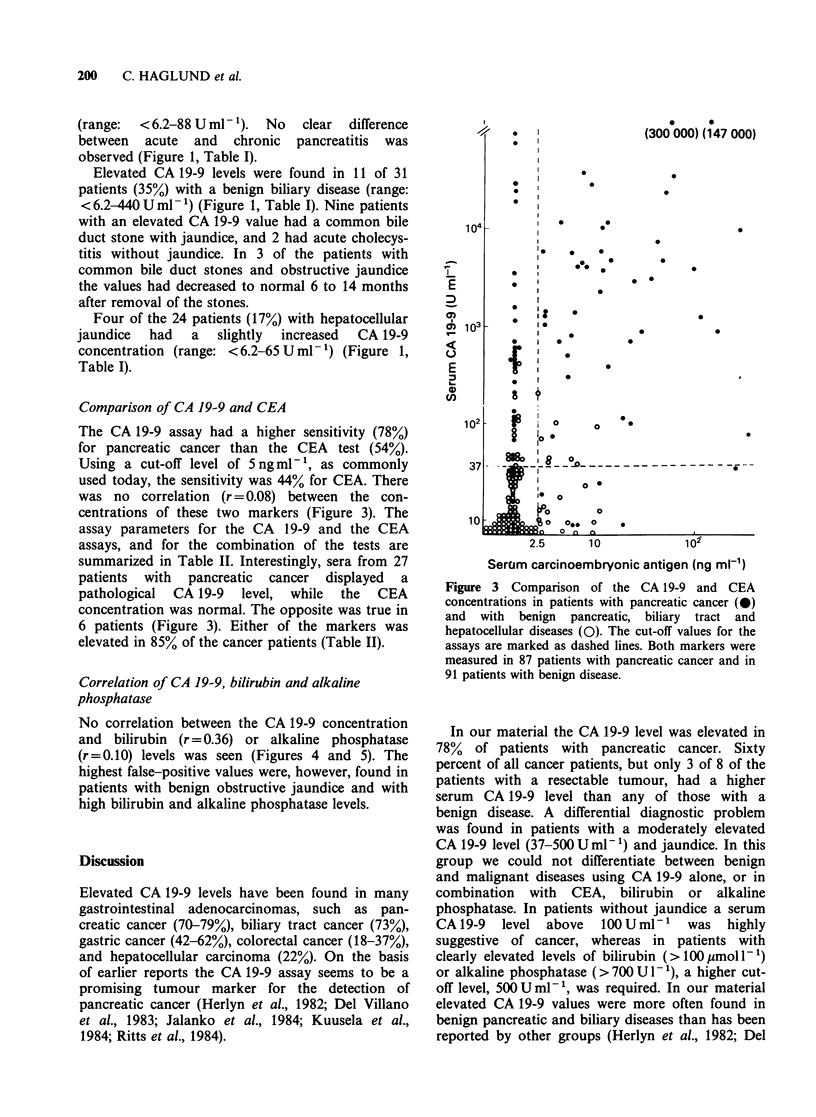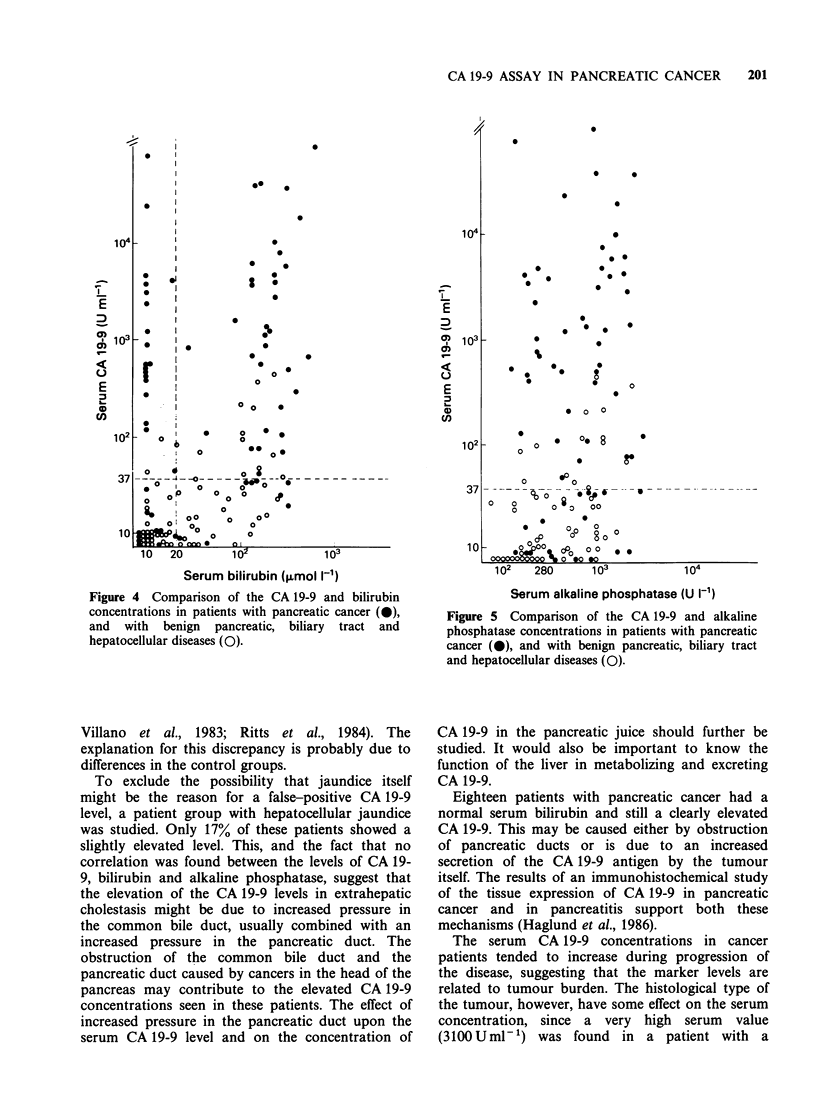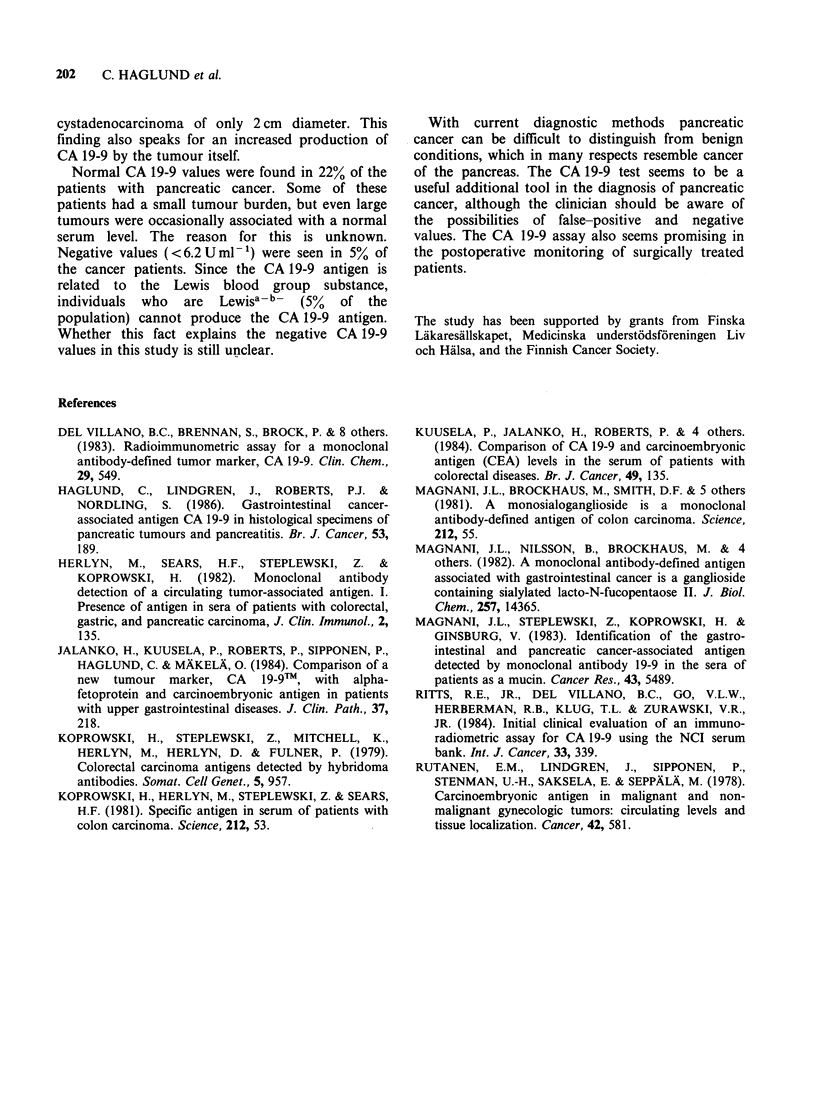# Evaluation of CA 19-9 as a serum tumour marker in pancreatic cancer.

**DOI:** 10.1038/bjc.1986.35

**Published:** 1986-02

**Authors:** C. Haglund, P. J. Roberts, P. Kuusela, T. M. Scheinin, O. Mäkelä, H. Jalanko

## Abstract

Serum concentrations of the CA 19-9 antigen were determined in 91 patients with pancreatic cancer and in 111 patients with benign pancreatic, biliary and hepatocellular diseases. The CA 19-9 concentration was above the cut-off limit (37 U ml-1) in 78% of the patients with pancreatic cancer and high levels (greater than 500 U ml-1) were seen in 56% of these patients. Elevated levels were also seen in benign diseases (22%), especially in patients with extrahepatic cholestasis (up to 440 U ml-1). Hepatocellular jaundice and pancreatitis were associated with normal values (84% of the patients), or with only slightly elevated CA 19-9 levels (up to 88 U ml-1). The CA 19-9 test can be useful as an additional diagnostic tool for the detection of pancreatic cancer. Preliminary results suggest that the CA 19-9 assay can be used in the monitoring of surgically treated patients.


*Br. J. Cancer* (1986), **53**, 197–202

### Evaluation of CA 19-9 as a serum tumour marker in pancreatic cancer

C. Haglund[1], P.J. Roberts[1], P. Kuusela[2], T.M. Scheinin[1], O. Mäkelä[2] & H. Jalanko[2]

[1]*Fourth Department of Surgery, Helsinki University Central Hospital and* [2]*Department of Bacteriology and Immunology, University of Helsinki, Helsinki, Finland.*

**Summary** Serum concentrations of the CA 19-9 antigen were determined in 91 patients with pancreatic cancer and in 111 patients with benign pancreatic, biliary and hepatocellular diseases. The CA 19-9 concentration was above the cut-off limit (37 U ml$^{-1}$) in 78% of the patients with pancreatic cancer and high levels (>500 U ml$^{-1}$) were seen in 56% of these patients. Elevated levels were also seen in benign diseases (22%), especially in patients with extrahepatic cholestasis (up to 440 U ml$^{-1}$). Hepatocellular jaundice and pancreatitis were associated with normal values (84% of the patients), or with only slightly elevated CA 19-9 levels (up to 88 U ml$^{-1}$). The CA 19-9 test can be useful as an additional diagnostic tool for the detection of pancreatic cancer. Preliminary results suggest that the CA 19-9 assay can be used in the monitoring of surgically treated patients.

The CA-19-9 radioimmunoassay is based on a monoclonal antibody, 1116 NS 19-9, raised against a human colorectal cell line (Koprowski *et al.*, 1979, 1981; Del Villano *et al.*, 1983). The antigenic determinant is a sialylated lacto-N-fucopentaose II, corresponding to a sialylated Lewis$^a$ blood group substance (Magnani *et al.*, 1981, 1982). This antigen is present in serum in a high molecular weight glycoprotein fraction, the mucin fraction (Magnani *et al.*, 1983).

Elevated serum CA 19-9 levels have previously been found in many different malignant diseases (Herlyn *et al.*, 1982; Del Villano *et al.*, 1983; Jalanko *et al.*, 1984; Kuusela *et al.*, 1984; Ritts *et al.*, 1984). The test seems especially promising for the detection of pancreatic cancer, 70–79% of these patients showing increased serum CA 19-9 concentrations (Del Villano *et al.*, 1983; Jalanko *et al.*, 1984; Ritts *et al.*, 1984). Moderately elevated values have also been found in patients with jaundice of benign origin (Jalanko *et al.*, 1984), but the association of elevated CA 19-9 values and jaundice has not been defined. Since most of the patients with pancreatic cancer also have jaundice, this question seems important. In this work, CA 19-9 levels were determined in patients with pancreatic cancer, and in patients with benign diseases representing differential diagnostic problems in clinical practice.

Correspondence: C. Haglund.
Received 29 July 1985; and in revised form, 10 October 1985.

### Patients and methods

### *Patients*

Serum samples were obtained from 91 patients with pancreatic cancer. Eight patients had a local resectable tumour, all other patients had either a locally spread or a metastasized tumour. The cancers included 2 islet cell carcinomas, 1 carcinoid tumour of the pancreas, 3 cystadenocarcinomas, 2 anaplastic carcinomas, 13 poorly differentiated ductal adenocarcinomas and 33 well to moderately differentiated adenocarcinomas. In 37 patients with an adenocarcinoma the exact degree of differentiation could not be determined. The serum samples were taken preoperatively. Repeated samples were obtained from three patients, who developed a recurrence after a radical operation, and from nine patients with a nonresectable tumour. Patients who had received radiotherapy to the pancreatic region were excluded from the present study.

A total of 56 patients had a benign pancreatic disease, including severe haemorrhagic pancreatitis (25 patients), non-haemorrhagic acute pancreatitis (19 patients), acute pancreatitis associated with pseudocyst formation (4 patients) and chronic pancreatitis (8 patients). Benign biliary tract diseases were found in 31 patients. These included 13 patients with common bile duct stones and cholestasis. One patient had cholestasis due to benign postoperative stenosis. Four patients had bile duct stones without jaundice, and 13 patients had gallbladder stones with an acute or chronic inflammation of the gallbladder. Control samples were obtained postoperatively from 3 of the

© The Macmillan Press Ltd., 1986

patients with benign obstructive jaundice after recovery, when their bilirubin values were normal. Hepatocellular jaundice was due to hepatic cirrhosis in 11 patients, and due to acute alcoholic hepatitis in 3 patients. Ten patients had viral hepatitis.

*Assays*

CA 19-9 antigen concentration was determined by a solid phase radioimmunoassay (Centocor, Malvern, PA, USA), using the recommended cut-off value of 37 U ml$^{-1}$ (Del Villano *et al.*, 1983). The serum samples were stored at −20°C or −70°C from 1 to 24 months before the CA 19-9 measurements. Carcinoembryonic antigen (CEA) was quantitated either by the double antibody radioimmunoassay, in which commercial anti-CEA antiserum (Dakopatts a/s, Copenhagen, Denmark) was used as the first antibody (Rutanen *et al.*, 1978), or by the Abbott-CEA-RIA Diagnostic Kit (Abbott, Weisbahn, FRG). These two tests have shown a good correlation (Jalanko *et al.*, 1984). A cut-off value of 2.5 ng ml$^{-1}$ was used.

*Routine laboratory data*

Serum bilirubin and serum alkaline phosphatase values were obtained from clinical records, when available. Cut-off values of 20 $\mu$mol l$^{-1}$ and 280 U ml$^{-1}$, respectively, were used.

### Results

*CA 19-9 in pancreatic cancer*

The range of serum CA 19-9 concentration in the 91 patients was <6.2–300 000 U m$^{-1}$ and the median value was 705 U ml$^{-1}$. Seventy-one of the patients with pancreatic cancer (78%) had a serum CA 19-9 concentration >37 U ml$^{-1}$ (Figure 1, Table I), and more than half (56%) of these patients had a concentration >500 U ml$^{-1}$ (Table II). High levels were found especially in patients with widely disseminated disease, but also 5 out of 8 patients with a resectable pancreatic tumour had elevated values (range: 34 – 12 500 U ml$^{-1}$). The concentration was increased in 25 out of 33 patients (76%) with a well to moderately differentiated adenocarcinoma and in 11 out of 15 patients (73%) with a poorly differentiated or an anaplastic carcinoma. All three patients with a cystadenocarcinoma had a clearly increased serum CA 19-9 concentration. Two patients with an islet cell carcinoma had a normal CA 19-9 level, while in one patient with a carcinoid tumour, the level was slightly elevated, 48 U ml$^{-1}$.

Changes in the serum CA 19-9 values were followed in 11 patients. In 2 surgically treated

**Figure 1** Serum CA 19-9 concentrations in patients with localized (■) and advanced (●) pancreatic cancer; and with acute (○) and chronic (△) pancreatitis; benign biliary tract diseases with (○) and without (□) jaundice and hepatocellular diseases. The cut-off value for the CA 19-9 test is marked as a dashed line.

patients the CA 19-9 level was elevated preoperatively, decreased after surgery and began to increase 4 and 8 months before appearance of symptoms or signs of recurrence. In the third patient, the preoperative value was 36 U ml$^{-1}$, it decreased after the operation, and increased above the cut-off level after the clinical detection of recurrence (Figure 2). The CA 19-9 concentration increased with tumour progression in 5 patients, who underwent by-pass surgery, and in 3 conservatively treated patients. The level decreased after by-pass surgery (from 2850 to 845 U ml$^{-1}$) in one patient with preoperative jaundice and a normal serum bilirubin by the time of control (6 weeks postoperatively).

*CA 19-9 in benign diseases*

Nine of 56 of the patients (16%) with pancreatitis had a slightly increased CA 19-9 concentration

**Table I** Serum CA 19-9 concentrations in patients with pancreatic cancer and with benign pancreatic, biliary tract and hepatocellular diseases, using various cut-off levels

| | | CA 19-9 | | | |
|---|---|---|---|---|---|
| *Diagnosis* | *No. tested* | *>37 U ml$^{-1}$* | *>100 U ml$^{-1}$* | *>300 U ml$^{-1}$* | *>500 U ml$^{-1}$* |
| Pancreatic cancer | 91 | 78% | 73% | 63% | 56% |
| Pancreatitis | 56 | 16% | 0% | 0% | 0% |
| Benign biliary disease | 31 | 35% | 23% | 6% | 0% |
| Hepatocellular jaundice | 24 | 17% | 0% | 0% | 0% |

**Table II** Assay parameters for the CA 19-9 and the CEA assays, and for the combination of the tests

| *Assay parameter* | *CA 19-9+*[a] | *CEA+*[b] | *CA 19-9+ or CEA+* | *CA 19-9+ and CEA+* |
|---|---|---|---|---|
| Sensitivity[c] | 78% | 54% | 85% | 47% |
| Specificity[d] | 78% | 76% | 62% | 92% |
| Predictive value[e] | 75% | 68% | 68% | 85% |

[a]CA 19-9+: >37 U ml$^{-1}$. [b]CEA+: >2.5 ng ml$^{-1}$. [c]Sensitivity = TP/(TP+FN). [d]Specificity = TN/(TN+FP). [e]Predictive value = TP/(TP+FP). TP = true positive; FN = false negative; TN = true negative; FP = false positive.

**Figure 2** CA 19-9 (a) and CEA (b) levels in three patients, who underwent pancreaticoduodenectomy for pancreatic cancer, and developed a recurrence. The arrows indicate the time of clinical verification of the recurrence.

(range: <6.2–88 U $ml^{-1}$). No clear difference between acute and chronic pancreatitis was observed (Figure 1, Table I).

Elevated CA 19-9 levels were found in 11 of 31 patients (35%) with a benign biliary disease (range: <6.2–440 U $ml^{-1}$) (Figure 1, Table I). Nine patients with an elevated CA 19-9 value had a common bile duct stone with jaundice, and 2 had acute cholecystitis without jaundice. In 3 of the patients with common bile duct stones and obstructive jaundice the values had decreased to normal 6 to 14 months after removal of the stones.

Four of the 24 patients (17%) with hepatocellular jaundice had a slightly increased CA 19-9 concentration (range: <6.2–65 U $ml^{-1}$) (Figure 1, Table I).

### *Comparison of CA 19-9 and CEA*

The CA 19-9 assay had a higher sensitivity (78%) for pancreatic cancer than the CEA test (54%). Using a cut-off level of 5 ng $ml^{-1}$, as commonly used today, the sensitivity was 44% for CEA. There was no correlation ($r = 0.08$) between the concentrations of these two markers (Figure 3). The assay parameters for the CA 19-9 and the CEA assays, and for the combination of the tests are summarized in Table II. Interestingly, sera from 27 patients with pancreatic cancer displayed a pathological CA 19-9 level, while the CEA concentration was normal. The opposite was true in 6 patients (Figure 3). Either of the markers was elevated in 85% of the cancer patients (Table II).

### *Correlation of CA 19-9, bilirubin and alkaline phosphatase*

No correlation between the CA 19-9 concentration and bilirubin ($r = 0.36$) or alkaline phosphatase ($r = 0.10$) levels was seen (Figures 4 and 5). The highest false-positive values were, however, found in patients with benign obstructive jaundice and with high bilirubin and alkaline phosphatase levels.

### Discussion

Elevated CA 19-9 levels have been found in many gastrointestinal adenocarcinomas, such as pancreatic cancer (70–79%), biliary tract cancer (73%), gastric cancer (42–62%), colorectal cancer (18–37%), and hepatocellular carcinoma (22%). On the basis of earlier reports the CA 19-9 assay seems to be a promising tumour marker for the detection of pancreatic cancer (Herlyn *et al.*, 1982; Del Villano *et al.*, 1983; Jalanko *et al.*, 1984; Kuusela *et al.*, 1984; Ritts *et al.*, 1984).

**Figure 3** Comparison of the CA 19-9 and CEA concentrations in patients with pancreatic cancer (●) and with benign pancreatic, biliary tract and hepatocellular diseases (○). The cut-off values for the assays are marked as dashed lines. Both markers were measured in 87 patients with pancreatic cancer and in 91 patients with benign disease.

In our material the CA 19-9 level was elevated in 78% of patients with pancreatic cancer. Sixty percent of all cancer patients, but only 3 of 8 of the patients with a resectable tumour, had a higher serum CA 19-9 level than any of those with a benign disease. A differential diagnostic problem was found in patients with a moderately elevated CA 19-9 level (37–500 U $ml^{-1}$) and jaundice. In this group we could not differentiate between benign and malignant diseases using CA 19-9 alone, or in combination with CEA, bilirubin or alkaline phosphatase. In patients without jaundice a serum CA 19-9 level above 100 U $ml^{-1}$ was highly suggestive of cancer, whereas in patients with clearly elevated levels of bilirubin (>100 $\mu$mol $l^{-1}$) or alkaline phosphatase (>700 U $l^{-1}$), a higher cut-off level, 500 U $ml^{-1}$, was required. In our material elevated CA 19-9 values were more often found in benign pancreatic and biliary diseases than has been reported by other groups (Herlyn *et al.*, 1982; Del

**Figure 4** Comparison of the CA 19-9 and bilirubin concentrations in patients with pancreatic cancer (●), and with benign pancreatic, biliary tract and hepatocellular diseases (○).

**Figure 5** Comparison of the CA 19-9 and alkaline phosphatase concentrations in patients with pancreatic cancer (●), and with benign pancreatic, biliary tract and hepatocellular diseases (○).

Villano *et al.*, 1983; Ritts *et al.*, 1984). The explanation for this discrepancy is probably due to differences in the control groups.

To exclude the possibility that jaundice itself might be the reason for a false–positive CA 19-9 level, a patient group with hepatocellular jaundice was studied. Only 17% of these patients showed a slightly elevated level. This, and the fact that no correlation was found between the levels of CA 19-9, bilirubin and alkaline phosphatase, suggest that the elevation of the CA 19-9 levels in extrahepatic cholestasis might be due to increased pressure in the common bile duct, usually combined with an increased pressure in the pancreatic duct. The obstruction of the common bile duct and the pancreatic duct caused by cancers in the head of the pancreas may contribute to the elevated CA 19-9 concentrations seen in these patients. The effect of increased pressure in the pancreatic duct upon the serum CA 19-9 level and on the concentration of CA 19-9 in the pancreatic juice should further be studied. It would also be important to know the function of the liver in metabolizing and excreting CA 19-9.

Eighteen patients with pancreatic cancer had a normal serum bilirubin and still a clearly elevated CA 19-9. This may be caused either by obstruction of pancreatic ducts or is due to an increased secretion of the CA 19-9 antigen by the tumour itself. The results of an immunohistochemical study of the tissue expression of CA 19-9 in pancreatic cancer and in pancreatitis support both these mechanisms (Haglund *et al.*, 1986).

The serum CA 19-9 concentrations in cancer patients tended to increase during progression of the disease, suggesting that the marker levels are related to tumour burden. The histological type of the tumour, however, have some effect on the serum concentration, since a very high serum value ($3100\,U\,ml^{-1}$) was found in a patient with a cystadenocarcinoma of only 2 cm diameter. This finding also speaks for an increased production of CA 19-9 by the tumour itself.

Normal CA 19-9 values were found in 22% of the patients with pancreatic cancer. Some of these patients had a small tumour burden, but even large tumours were occasionally associated with a normal serum level. The reason for this is unknown. Negative values ($<6.2\,U\,ml^{-1}$) were seen in 5% of the cancer patients. Since the CA 19-9 antigen is related to the Lewis blood group substance, individuals who are Lewis$^{a-b-}$ (5% of the population) cannot produce the CA 19-9 antigen. Whether this fact explains the negative CA 19-9 values in this study is still unclear.

With current diagnostic methods pancreatic cancer can be difficult to distinguish from benign conditions, which in many respects resemble cancer of the pancreas. The CA 19-9 test seems to be a useful additional tool in the diagnosis of pancreatic cancer, although the clinician should be aware of the possibilities of false–positive and negative values. The CA 19-9 assay also seems promising in the postoperative monitoring of surgically treated patients.

The study has been supported by grants from Finska Läkaresällskapet, Medicinska understödsföreningen Liv och Hälsa, and the Finnish Cancer Society.

**References**

DEL VILLANO, B.C., BRENNAN, S., BROCK, P. & 8 others. (1983). Radioimmunometric assay for a monoclonal antibody-defined tumor marker, CA 19-9. *Clin. Chem.*, **29**, 549.

HAGLUND, C., LINDGREN, J., ROBERTS, P.J. & NORDLING, S. (1986). Gastrointestinal cancer-associated antigen CA 19-9 in histological specimens of pancreatic tumours and pancreatitis. *Br. J. Cancer*, **53**, 189.

HERLYN, M., SEARS, H.F., STEPLEWSKI, Z. & KOPROWSKI, H. (1982). Monoclonal antibody detection of a circulating tumor-associated antigen. I. Presence of antigen in sera of patients with colorectal, gastric, and pancreatic carcinoma, *J. Clin. Immunol.*, **2**, 135.

JALANKO, H., KUUSELA, P., ROBERTS, P., SIPPONEN, P., HAGLUND, C. & MÄKELÄ, O. (1984). Comparison of a new tumour marker, CA 19-9$^{TM}$, with alpha-fetoprotein and carcinoembryonic antigen in patients with upper gastrointestinal diseases. *J. Clin. Path.*, **37**, 218.

KOPROWSKI, H., STEPLEWSKI, Z., MITCHELL, K., HERLYN, M., HERLYN, D. & FULNER, P. (1979). Colorectal carcinoma antigens detected by hybridoma antibodies. *Somat. Cell Genet.*, **5**, 957.

KOPROWSKI, H., HERLYN, M., STEPLEWSKI, Z. & SEARS, H.F. (1981). Specific antigen in serum of patients with colon carcinoma. *Science*, **212**, 53.

KUUSELA, P., JALANKO, H., ROBERTS, P. & 4 others. (1984). Comparison of CA 19-9 and carcinoembryonic antigen (CEA) levels in the serum of patients with colorectal diseases. *Br. J. Cancer*, **49**, 135.

MAGNANI, J.L., BROCKHAUS, M., SMITH, D.F. & 5 others (1981). A monosialoganglioside is a monoclonal antibody-defined antigen of colon carcinoma. *Science*, **212**, 55.

MAGNANI, J.L., NILSSON, B., BROCKHAUS, M. & 4 others. (1982). A monoclonal antibody-defined antigen associated with gastrointestinal cancer is a ganglioside containing sialylated lacto-N-fucopentaose II. *J. Biol. Chem.*, **257**, 14365.

MAGNANI, J.L., STEPLEWSKI, Z., KOPROWSKI, H. & GINSBURG, V. (1983). Identification of the gastro-intestinal and pancreatic cancer-associated antigen detected by monoclonal antibody 19-9 in the sera of patients as a mucin. *Cancer Res.*, **43**, 5489.

RITTS, R.E., JR., DEL VILLANO, B.C., GO, V.L.W., HERBERMAN, R.B., KLUG, T.L. & ZURAWSKI, V.R., JR. (1984). Initial clinical evaluation of an immuno-radiometric assay for CA 19-9 using the NCI serum bank. *Int. J. Cancer*, **33**, 339.

RUTANEN, E.M., LINDGREN, J., SIPPONEN, P., STENMAN, U.-H., SAKSELA, E. & SEPPÄLÄ, M. (1978). Carcinoembryonic antigen in malignant and non-malignant gynecologic tumors: circulating levels and tissue localization. *Cancer*, **42**, 581.